# The plasticity of ageing and the rediscovery of ground-state prevention

**DOI:** 10.1007/s40656-021-00414-6

**Published:** 2021-05-04

**Authors:** Alessandro Blasimme

**Affiliations:** Department of Health Sciences and Technology, Swiss Federal Institute of Technology (ETH Zurich), Zurich, Switzerland

**Keywords:** Ageing, Explanatory frameworks, Plasticity, Health span, Compression of morbidity, Geroprotectors, Senolytics

## Abstract

In this paper, I present an emerging explanatory framework about ageing and care. In particular, I focus on how, in contrast to most classical accounts of ageing, biomedicine today construes the ageing process as a modifiable trajectory. This framing turns ageing from a stage of inexorable decline into the focus of preventive strategies, harnessing the functional plasticity of the ageing organism. I illustrate this shift by focusing on studies of the demographic dynamics in human population, observations of ageing as an intraspecifically heterogenous phenotype, and the experimental manipulation of longevity, in both model organisms and humans. I suggest that such an explanatory framework about ageing creates the epistemological conditions for the rise of a peculiar form of prevention that does not aim to address a specific condition. Rather it seeks to stall the age-related accumulation of molecular damage and functional deficits, boosting individual resilience against age-related decline. I call this preventive paradigm “ground-state prevention.” While new, ground-state prevention bears conceptual resemblance to forms of medical wisdom prominent in classic Galenic medicine, as well as in the Renaissance period.

## Introduction

Over the past century, medical progress has led to some of the most remarkable health improvements in the evolutionary history of the human species. Thanks to the ability of medicine to prevent and treat communicable diseases, and its increased capacity to diagnose and manage chronic conditions, humans today can expect to live substantially longer lives than any previous generation. Such circumstances have however had a less welcome consequence. According to recent estimates, about half of the global burden of disease—understood in terms of disability-adjusted life years (DALYs) per 1000 adults—is currently age-related (Chang et al., 2019). At a global level, these figures have been improving for the last 20 years (ivi). Still, in absolute terms, the burden of age-related disease remains high, and great disparities exist between more and less developed countries, the latter facing a much greater burden. These observations explain why ageing is a major global public health issue attracting considerable research and policy interest worldwide.

However in many respects, ageing remains an elusive phenomenon (Blasimme, Boniolo, & Nathan, 2021). Philosophers and historians, along with social scientists and bioethicists, have long engaged with foundational conceptual issues related to ageing (Garson, 2021; Green & Hillersdal, 2021; Jecker, 2021; Sholl, 2021). In recent times, biology and medicine have greatly contributed to this debate, in an effort to define ageing from a scientific point of view, clarify its driving molecular mechanisms, and establish the legitimacy of clinical interference with the ageing process. These efforts have resulted in several biomedical theories of ageing (Jin, 2010; Bengtson and Settersten, 2016). Nevertheless, there is no consensus on what ageing is, or the medical implications of different ontological accounts of the ageing process.

To illustrate how evolving views on the concept of ageing correspond with specific ideas about care and prevention, in this paper, I discuss an emerging explanatory framework about ageing and care. In particular, I focus on how, in contrast to most classical accounts of ageing, contemporary biomedicine constructs the ageing process as a modifiable trajectory. In doing so, it transforms ageing from a stage of inexorable decline to the focus of preventive strategies, harnessing the functional plasticity of ageing organisms. Studies on the demographic dynamics of the human population, observations of ageing as an intraspecifically heterogenous phenotype, and advances in the experimental manipulation of longevity in both model organisms and humans, have made it scientifically plausible to look at ageing as a plastic phenotype. The account of prevention associated with the plasticity of ageing does not treat ageing as an ailment to be averted. Rather it aims to delay age-related accumulation of molecular damage and functional deficits, boosting individual resilience against age-related decline. I propose to call this preventive paradigm *ground-state prevention* and show that, while new, it bears conceptual resemblance with medical wisdom prominent in classic Galenic medicine, as well as in the Renaissance period.

The aim of this paper is to shed light on novel conceptualizations of ageing and care that are taking shape at the intersection of the life sciences, geriatric medicine, and public health. Clarifying such accounts of ageing (both epistemic and normative) is important, as this provides a philosophical appraisal of their conceptual and socio-ethical foundations.

The paper is structured as follows. Section 2 sets the stage by clarifying the idea of explanatory frameworks of ageing and care. Sections 3 illustrates the received view of ageing as unmodifiable trajectory. Section 4 focuses on contemporary conceptualizations of ageing in biomedicine, showing how the medical control of longevity and health span became a plausible medical objective. Section 5 explains the notion of ground-state prevention as a nascent paradigm in geriatrics and public health. Finally, Sect. 6 concludes with reflections on the implications of such conceptual shifts for the philosophy of science and medicine.

## Background: explanatory frameworks of ageing

In his 1957 essay*On Ageing and Old Age*, Mirko Grmek provided an authoritative historical exposition of different accounts of ageing, from antiquity to the twentieth century (Grmek, 1957).^1^ According to Grmek, ageing has generally been understood as a “progressive and irreversible changing of the structures and functions of living systems” (Grmek, 1957, 60). In classic medicine, such process was attributed either to a “loss of something important for the maintenance of life [or] as the accumulation, the surplus of something that is deleterious” (ibid.).

These two hypotheses are still present in contemporary accounts of ageing. Modern biological theories of ageing have been divided into two main families: programmed-ageing theories and error theories (Jin, 2010).

*Programmed-ageing theories* stress that ageing follows a biologically defined trajectory, leading to progressive functional decline. They include the following hypotheses.

1. A species’ life span is inversely correlated with that species’ metabolic rate. Ageing depends on how fast an organism uses up a fixed quantity of metabolic potential (Hulbert et al., 2007; Pearl, 1928b; Rubner, 1908; Sohal, 1986).
2. Ageing is determined by increased genomic instability, that is, the progressive accumulation of genetic mutations resulting in defects in DNA replication, and affecting gene expression by down- or up-regulation of DNA transcription.
3. Ageing is mediated by highly-conserved signaling pathways which play a role in hormonal regulation.
4. Ageing is due to the natural deterioration of the neuroendocrine and immune system eroding respectively an organism’s capacity to maintain homeostasis and to cope with infectious diseases, inflammation, cancer, and other age-related conditions.

*Error theories* view ageing as the result of damage accumulation due to exposure to environmental hazards. They explain this phenomenon through a number of molecular mechanisms. At a general level, cells and tissue wear out over time, leading to organ dysfunction and eventually death. A more specific explanation connects ageing to the age-dependent accumulation of cross-linked proteins that damage cells, tissues, and organs. A more current version of this hypothesis holds that accumulated cellular damage is a result of free radicals (a by-product of cellular metabolism due to diet, inflammation, radiation, and pollution) acting on cells’ macromolecules. Finally, ageing is explained by the accumulation of unrepaired DNA damage over time, leading to somatic DNA mutations or an excess of cells entering senescence, that is, a state of cellular dormancy triggered by the presence of unpreparable DNA alterations and linked to organ dysfunction.

Other categorizations of ageing theories have been proposed. They differ according to the relevance attributed to different explanatory levels, namely the evolutionary, molecular, cellular, and system levels (Weinert & Timiras, 2003). Evolutionary theories of ageing, in particular, include the disposable soma theory, according to which deleterious mutations past reproductive age are not selected against (Kirkwood & Holliday, 1979); and the antagonistic pleiotropy theory, stating that genes beneficial for the young are instead harmful to the old (Garson, 2021; Williams, 1957).

This rapid excursus reveals what Grmek noted more than 50 years ago: that “the causes of ageing have not yet been scientifically explained, nor has there been any answer to the question of what is essential and primary in the process of senile involution” (cit. 60). What we have observed so far is a set of competing theoretical accounts and scientific hypotheses about ageing and its causes.

For analytical purposes it will be convenient to, first of all, clarify the epistemological nature of such conceptual accounts. Echoing the language of the cited sources, I have so far used a number of different expressions to refer to such conceptual accounts as theories, hypotheses, or simply understandings of ageing.

Different scientific understandings of ageing, however, are not to be taken as full-blown theories, either in a syntactic or semantic sense. They do not state axioms or laws of nature, or allow law-like or probabilistic generalizations. They are not classes of models and are not amenable to falsification (Frigg, 2006; Thompson, 1989; Van Fraassen, 1989). Rather they are best understood as *explanatory frameworks* (Blasimme et al., 2013). They provide conceptual perspectives on ageing, offering “overarching patterns of explanation that subsume a variety of mechanisms and a multiplicity of diverse data under a common gaze” (Blasimme et al., 2013, 381). Explanatory frameworks are theoretical in nature, but their function is not to represent hegemonic conceptual accounts of a biological phenomenon. Different explanatory frameworks can coexist and evolve to incorporate elements of one another over time. This inherently pluralistic character of explanatory frameworks, as opposed to the more monistic nature of causal mechanisms, is a well-established epistemological trait of contemporary biomedicine—one that finds confirmation in the scientific literature on the physiology of ageing (Weinert & Timiras, 2003). The function of explanatory frameworks is to drive the formulation of scientific hypotheses and the discovery of biological mechanisms, attributing explanatory relevance to some mechanisms or levels of explanation rather than others. In words of Grmek, explanatory frameworks of ageing indicate “what is essential and primary in the process of senile involution” (Grmek, 1957, 64). Explanatory frameworks exist at a higher level of generality than sheer biological mechanisms (Machamer et al., 2000). Therefore, while they play a fundamental explanatory role, such theoretical accounts do not dispense with mechanisms as the fundamental explanatory level of biological phenomena. Rather, they promote the discovery of mechanisms and their selection as relevant explanatory aspects of the phenomenon at stake—ageing in our case.

In the next section, I discuss present-day explanatory frameworks which mark a departure from the previously common view of ageing as an inescapable trajectory of decline. Contemporary biomedicine instead stresses functional plasticity as an ontological feature of the ageing process, regarding ageing as a more plastic and modifiable process than previously hypothesized. Speaking about ageing in terms of functional plasticity does not serve explanatory purposes alone. It also incorporates and supports normative considerations about what we owe to elders in terms of care.

## Stiff senescence: the received view

The scientific debate about the physiology of human ageing and its relation to an increased probability of pathological affections has long been dominated by a rather pessimistic outlook. Since antiquity, ageing has been seen as a process of decline implying all sorts of diseases, including the degradation of mental faculties. Aristotle, who attributed ageing to a progressive cooling of the body, also correlated this process with a degradation of the moral temperament of old people(Woodcox, 2018). This view resonates with more modern accounts of ageing, stressing increased disease incidence and disability as fundamental and inevitable traits of old age.^2^ Age-related decline, in this respect, is not only the effect of disease. It also occurs as a result of physiological—not only pathological—processes.

A clear distinction between normal and pathological degeneration has never been easy to draw. Ancient Greeks and Romans attributed senescence to the same fundamental mechanisms that cause disease (i.e., improper mix of humours, or dyskrasia), and were therefore inclined to consider ageing as a pathological state, notably, an *incurable* one—as in the famous dictum by Seneca ‘*senectus insanabilis morbus’* (Grmek, 1957, 61). Galen did not consider ageing as a disease. He acknowledged the increased incidence of disease in old age, and considered it unavoidable and hardly open to modifying interventions. In modern times, evolutionary theories of ageing have further reinforced physiological views (see above). Such theories centre around adaptationist explanations of why, after reproductive age, natural selection no longer exerts strong pressure on the maintenance of fitness, thus rendering an organism naturally more vulnerable as it ages. If a pathological account equating ageing to a disease is somewhat counterintuitive, Grmek reminds us that the idea of physiological ageing is not without its own philosophical problems, as age-related decline is rarely seen as the simultaneous degradation of all organs and bodily systems. It is therefore hard to consider the functional manifestation of age-dependent decline separate from a specific pathological affection occurring in this or that organ. I will return to these considerations in Sect. 5. For the time being, suffice it to say that a somewhat fatalistic view of ageing as an unavoidable, untreatable, and unmodifiable trajectory of decline has survived to modern times. It is against this backdrop that geriatrics has flourished as a medical speciality aimed at “doing something more” for elders, from a medical point of view. A characteristic feature of this nascent speciality in the early twentieth century was its insistence on reverting to precisely these inherited views about ageing, regarding old age as a legitimate site for medical intervention.

Marjory Warren, a pioneering figure of the British geriatrics movement, is credited as an initiator of this paradigm shift. In 1946 Warren wrote a pragmatic manifesto in *The Lancet* aimed at calling attention to the preventable effects of medical neglect of older patients. She vividly describes the consequences of this lack of attention as a rapid functional and psychological decline of elders (“untreated cases”) to a “miserable state, dull, apathetic, helpless and hopeless” (Warren, 1946, 6407). In this way, Warren constructs the premise for a new conception of age-related decline amenable to what she calls “rehabilitation”. This paradigm shift is sustained by a militant attention to the otherwise neglected needs of elders. In particular, it draws attention to the iatrogenic effects of practices like unnecessary hospitalization and excessive bedrest, and to the lack of cognitive stimulation, socialization, healthy diet, and physical activity, as key determinants of physical and mental health during senescence.

The history of geriatrics in the last decades has been marked by the piecemeal development of these initial ideas. As a consequence, geriatric medicine has articulated a model of care centred around ageing as a modifiable trajectory, and thus a legitimate site of medical care and prevention (Blasimme et al., 2021). This new focus weaves together ethical considerations about what we owe to elders in terms of medical care, and new explanatory frameworks about ageing and its relation to disease.

## Ageing as a plastic phenotype

Today, ageing is no longer understood in terms of incidence of disease and disability per se. Yale geriatricians Mary Tinetti and Terri Fried, for instance, have elaborated a view of geriatrics that moves altogether beyond the notion of disease as the focus of medical care (see Sect. 5). Instead, contemporary explanatory frameworks of ageing revolve around decline in an organism’s ability to respond to stress, leading to functional degradation, pathological states, and death (Weinert & Timiras, 2003). We have already encountered this idea of a fixed amount of livelihood consumed as an organism ages, in the metabolic account of ageing (see above—Sect. 2). This explanatory framework also resonates with George Canguilhem’s view of health and decline over the life course:

The life of the individual is, from its beginnings, the reduction of life’s powers. Because health is not a constant of satisfaction, but the a priori of the power to master perilous situations, this power uses itself up in mastering successive perils (Canguilhem, 2012, 65-66).

Currently, biomedical research interprets decline as a function of the erosion of organ reserve and intrinsic capacity. In a 1980 landmark paper in The New England Journal of Medicine, James Fried speaks of organ reserve as the capacity of organs to compensate for reduction of internal homeostasis. According to Fried, this capacity is highly redundant in the organs of the young, but wanes out over time, thus reducing an organism’s ability to restore homeostasis “when it is deranged by external threat” (Fried, 1980, 130). External perturbations in the context of severe reduction of organ reserve lead to death even in the absence of a clearly definable pathological state—a notion that resonates with the century-old idea of *natural death* occurring in very old age. On the other hand, chronic diseases typically affecting elders today can be seen “as problems of accelerated loss of organ reserve” (Fried, 1980, 132). Intrinsic capacity is a contemporary version of the idea of organ reserve promoted by the World Health Organization (WHO) in a public health and health promotion context (Beard et al., 2016). Intrinsic capacity is defined as the composite of an individual’s physical and mental capacities (WHO, 2015). It comprises a set of functional domains including psychological disposition, vitality (energy metabolism), sensory capacity, locomotion, and cognition (Cesari et al., 2018). Intrinsic capacity is influenced by genetic as well as health-related characteristics, such as disease and risk thereof. The combination of intrinsic capacity, environmental factors, and personal characteristics (such as sex, gender, ethnicity, occupation, education, and socio-economic status) determines an individual’s functional ability throughout the life course and affects well-being in older age (WHO 2015; Cesari et al., 2018; Blasimme, 2020a, b).

While concepts such as organ reserve and intrinsic capacity might appear to be fixed background conditions of ageing, they are to be understood in the context of an interventionist account of ageing. The idea that ageing is a process amenable to interventions aimed at slowing it down or influencing its trajectory owes to specific epistemological paradigms that emerged between the late 1970s and early 1980s. These include the compression of morbidity and the experimental control of life- and health span.

### Compressing morbidity and senescence

Different individuals age differently. Therefore, not everyone experiences a similar trajectory, and being of a given age does not affect the capacity of different individuals to function in the same way. In 1980, James Fries introduced the notion that through physical exercise, weight control, and “growth in personal autonomy and personal responsibility for health,” one can realize a compression of morbidity and senescence (Fried, 1980, 133). The human life span does not seem amenable to substantial improvement (Barbi et al., 2018), and many age-related conditions are not preventable. However, “the end of the period of adult vigor” (Fried, 1980, 134) can be postponed by delaying the onset of chronic illness, thus compressing morbidity towards the final years of life. Senescence can be compressed as well, preventing premature organ dysfunction that in most cases results from disuse, rather than overuse, of one’s faculties. This idea is summarized by the common geriatric adage “use it or lose it”. The emphasis is not on longevity but rather on improving the so-called health span, adding years of good health to the human life span by compressing disease and functional decline to the end of life.

Discussing this idea, Fried speaks of the “modifiability or ‘*plasticity’* of aging” (ibid., emphasis mine). He further states:

Variation between healthy persons of the same age is far greater than the variation due to age; age is a relatively unimportant variable, and training in marathon running is clearly more important than age. (ibid.)

Personal responsibility for health is clearly relevant in this explanatory framework. However, it should be coupled with health policies aimed at removing environmental hazards and developing incentives “to encourage […] the exercise of personal choice” (ibid.). This vision anticipates the health promotion turn in geriatrics, epitomized by the healthy ageing program developed in 2015 by the WHO.

Intrinsic capacity is of paramount importance to healthy ageing. In this paradigm the focus is on the longitudinal evaluation of health trajectory, rather than on understanding an individual’s health state based on the enumeration of diseases and ailments affecting her (Cesari et al., 2018). Research on the functional aspects of ageing and their relation to specific risk factors began in the late nineties (Stuck et al., 1999) and formed the evidence base for our present-day understanding of intrinsic capacity from a clinical point of view. According to current research on the determinants of age-related functional decline, the construct of intrinsic capacity encapsulates five functional domains (Cesari et al., 2018): cognition (memory, intelligence, problem solving), locomotion (balance, muscle strength, and gait speed), sensory functions (vision, hearing), psychological functions (mood, sociality) and vitality (metabolism, diet).

Functional deterioration in one or more the above domains leads to a higher risk of negative health outcomes, incidental functional loss, and care dependency. For instance, decline in locomotory function, mostly due to decreased use over time, results in higher risks of falls, hospitalization, disability, and dependency. Issues in sensory functions like hearing correlate with risk of dementia (Livingston et al., 2017). Depressive symptoms, frequent in elders despite not meeting the nosological criteria for clinical depression, correlate with negative health outcomes among the elderly (Cesari et al., 2018).

However, all five functional domains are amenable to preventive interventions aimed at increasing intrinsic capacity, or at least protecting it from degradation. Preferred routes of intervention include healthy dietary habits, physical activity, and socialization. Regular physical activity protects against decline in locomotory function, and can reduce frailty (World Health Organization, 2015). Caloric restriction has been shown to considerably slow the onset of age-related disease, contributing to the compression of morbidity and senescence (Flanagan et al., 2020; Fontana & Klein, 2007). Numerous studies underway are searching for effective strategies to boost cognition (Blasimme, 2020a; National Academies of Sciences, 2017). Interventions on environmental factors, designed to prevent the effects of functional decline, are also being investigated. For instance, adapting the design of urban spaces is considered important for fall prevention, removing barriers for people with limited mobility, as well as increasing opportunity for social exchange and interaction.

From an epistemological point of view, a clinical paradigm aimed at improving functional trajectories depends on a view of ageing as a plastic phenotype. To the best of my knowledge, no explicit reference is made in this domain to the debate on phenotypic plasticity in evolutionary biology (Pigliucci, 2001; Pigliucci et al., 2006), despite the relevance of evolutionary considerations in biological theories of ageing. However, it cannot be ruled out that this debate at least indirectly influenced explanatory frameworks of ageing centered around functional plasticity. In particular, the work of theoretical biologist Mary Jane West-Eberhard on the evolutionary role of the environment acting on plastic phenotypes, resonates with the idea of multiple ageing phenotypes resulting from different trajectories of adaptation to their surrounding environment (West-Eberhard, 2003).

### The experimental control of longevity and health span

So far, I have described an explanatory framework of ageing resulting in a specific clinical approach to the improvement of functional trajectories—one based on *lifestyle* and *health promotion* interventions. However, as I explained in Sect. 2, explanatory frameworks should be seen as broad conceptual spaces that remain open to further articulation, and that may incorporate different mechanistic accounts of the phenomenon at stake. This consideration also applies to the clinical aspects of explanatory frameworks. In this section, I illustrate how functional plasticity has driven research into ageing as the site of *pharmacological* interventions aimed at protecting individual resilience against the effects of ageing.

Over the last hundred years, molecular biology has made substantial progress in the experimental control of longevity in simple model organisms. Early attempts at extending the life span of living organisms date back to Raymond Pearl’s experiments on fruit flies in the 1920s (Pearl, 1928a). By the 1960s, the relation between genotype, environment, and longevity was an established scientific fact (Parsons, 1966). The 1970s marked the beginning of biogerontological studies elucidating the genetic determinants of longevity in Nematodes (Klass, 1977; Klass & Hirsh, 1976).^3^ In recent years, research has focused on translating this body of knowledge and experimental practices to the area of human ageing (Flatt & Partridge, 2018; Niccoli & Partridge, 2012; Rando & Chang, 2012). Nowadays, research into the molecular underpinnings of human ageing has also embraced a logic of plasticity:

Once thought of as an inexorable, complex and lineage- specific process of accumulation of damage, ageing has turned out to be influenced by mechanisms that show strong evolutionary conservation. (Niccoli & Partridge, 2012, R741)

Despite the fact that aging appears to be inexorable, with the ultimate result being the death of the organism, it is incontrovertible that life span itself can be experimentally manipulated. (Rando & Chang, 2012, 46)

In particular, the role of the IIS (nutrient-sensing insulin/insulin-like growth factor)/TOR (Target of Rapamycin) signaling pathway in longevity, but also in the extension of the health span in model organisms (Fontana et al., 2010) and in humans (Slagboom et al., 2011; Wheeler & Kim, 2011), has been demonstrated.

Dietary restriction is among the best understood methods for prolonging longevity and promoting individual health span (Omodei & Fontana, 2011). However, great efforts are being devoted to exploring the potential of molecules such as rapamycin, metformin, and their natural equivalents, for targeting metabolic pathways implicated in healthy longevity (Aliper et al., 2017; Baumann, 2018; Bellantuono, 2018; Ehninger et al., 2014; Huffman et al., 2016; Martin-Montalvo et al., 2013; Moskalev et al., 2016; Piskovatska et al., 2019; Trendelenburg et al., 2019; Xu et al., 2018).

This class of drugs does not focus on treating any particular condition. Rather the aim is to intervene on cellular pathways that influence ageing across a broad spectrum of mechanistic levels. Studies on the metabolic determinants of ageing have shown their importance for all biological and molecular hallmarks of ageing, from genomic instability to epigenetic alterations, and from mitochondrial dysfunction to cellular senescence and stem cell exhaustion (López-Otín et al., 2013, 2016). These drugs, increasingly referred to as *geroprotectors*, do not target any clearly defined pathological alteration, but rather exert a protective function (Fontana et al., 2014; Moskalev et al., 2016; Piskovatska et al., 2019; Trendelenburg et al., 2019).

I have presented two clinically diverging versions of the same explanatory framework focused on the plasticity of ageing. One is based on individual health choices and socio-environmental conditions of ageing; the other intends to recapitulate the expected outcomes of such interventions on healthy longevity through pharmacological means.

Other than viewing ageing in terms of functional plasticity, these two accounts share a novel understanding of prevention in the domain of ageing, as I explain in the next section.

## Ground-state prevention

Disease is considered the most fundamental unit of analysis in medicine. The definition of disease is the object of ongoing philosophical debate between naturalistic, constructivist, and instrumentalist accounts (Boorse, 1975; Caplan & McCartney, 2004; Nordenfelt, 1995, 2007; Wakefield, 1992). While there is no consensus on the concept of disease, the notion of disease is central to medicine. Until recently, the function of disease as the focus of medicine has gone unchallenged, due to its intuitive appeal as an obviously plausible target of medical intervention. However, the complexity of most age-related ailments, the fact that elders are affected by concomitant conditions (multimorbidity), and the fact that a great number of age-related symptomatic states escape clear nosological determination, have led some to question the utility of disease as the central focus of medical care. In a provoking 2004 paper on the “end of the disease era,” Tinetti and Fried explicitly criticized disease-centric medicine (Tinetti & Fried, 2004). They instead propose a more individualized approach that revolves around the clinical trade-offs necessary to manage a complexity of concomitant affections, and that takes into account patient preferences and expectations about such decisions. Instead of treating each disease individually, medicine should strive to treat an individual patient’s unique combination of diseases, and the way they affect physical and psychological functioning, as well as daily activities, goals, and life plans. The focus is less on discrete pathologies and survival, than on the reality of how an individual organism becomes diseased and weakened over time. This view, rather than being centered around an objectifying understanding of disease states, appeals to a broader notion of well-being as the core of any medical act. In particular, it shifts the focus of medical decision-making away from simply the functioning of discrete organs or bodily systems, to include the preferences, values, and priorities of patients (Canevelli et al. 2021).

The post-disease paradigm advanced by Tinetti and Fried is an example of a very general explanatory framework: one that considers multimorbidity, as opposed to discrete pathological states, to be the central focus of geriatric medicine. In this account, the health of the ageing person is best understood as the result of multiple concomitant pathologies. Medical decision making must therefore engage with this clinical complexity, striking a balance between the professional duty to cure the patient, and his or her preferences about treatment and outcomes. From a clinical point of view, this view takes a diachronic, life course perspective as to how an organism is affected by pathological states throughout its life, and how this progressively erodes resilience, reserves, and intrinsic capacity.

In contrast, understanding health as the absence of disease is not compatible with the idea of measuring and protecting functional trajectories as the focus of geriatric care. From an epistemological point of view, this vision clearly resonates with the explanatory framework of ageing as a plastic phenotype (Cesari et al., 2016). For researchers working on intrinsic capacity, the disease construct is inadequate for capturing how an individual fares in her environment in functional terms (Cesari et al., 2018). Function, not disease, is the object of care in this clinical perspective on ageing. The same applies to the molecular version of the explanatory framework. Researchers in this domain are not interested in linking alterations in metabolic pathways to the manifestation of a given discrete pathology. Their focus is rather on the role of those pathways in maintaining organ functionality over time. This, in turn, can translate into the delayed onset and slowing down of multiple diseases of old age (Bellantuono, 2018). The longitudinal focus of intrinsic capacity emphasizes prevention over reaction “even in the absence of a specific clinical phenotype” (Cesari et al., 2018, 1654).

If we look at those epistemological and clinical stances from the point of view of preventive medicine, is difficult to fit the objective of boosting an organism’s resilience to age-related decline into the primary, secondary, and tertiary prevention scheme (Tulchinsky & Varavikova, 2014, chap. 2). Primary prevention targets risk factors, with the aim of avoiding a specific disease before it occurs. Secondary prevention aims at preventing or reducing damage in individuals already exposed to risk factors or disease, but without clinical manifestations, symptoms, or dysfunctions (typically through regular disease-specific screenings). Finally, tertiary prevention concerns individuals who already have a disease or injury, and aims at rehabilitation or minimization of disease progression and adverse outcomes. The prevention of age-related functional decline—either by lifestyle, health promotion, or pharmacological interventions—does not correspond with any of the three levels of preventive medicine. For this reason, I propose to call this family of preventive approaches *ground-state prevention*. With this expression, I intend to capture the distinctive epistemological features of this form of preventive medicine. First, the fact that interventions do not target a specific risk factor. Second, the absence of a discrete pathological state as the focus of the intervention. And finally, the absence of a quantifiable health outcome as the benchmark of the intervention. This last point in particular may appear counterintuitive and calls for clarification. The objective of ground-state prevention is not living longer, or reaching the current average human life span. Rather it can be understood as gaining healthy life-years. Ground-state prevention does not aim at a given health span considered normal or species-typical. In this respect, while it is individual health spans that ground-state prevention seeks to improve, resilience-related outcomes are best measured in aggregate terms—that is, as prevalence of age-related functional measures such as frailty, multimorbidity, and the burden of age-related diseases. From the perspective of ground-state prevention, it makes sense to look at age in correlation with increased morbidity. However, it is not entirely warranted to see ageing itself as a risk factor, in light of the multiple realizability of ageing in terms of functional trajectories and well-being.

Ground-state prevention, while anchored in a novel explanatory framework of ageing, is not an entirely new idea. This approach recalls several century-old medical prescriptions about the preservation of personal health.

Medicine has long promoted healthy habits without the aim of preventing specific diseases, but rather with a focus on maintaining well-being throughout life. Classical medical treatises stress the negative effects of an excessively rich diet on quality of life and morbidity in old age (Schäfer, 2005). Lack of physical activity and an unbalanced diet were recognized as detrimental by Hippocrates, in his *Regime* or *De Diaeta*, and by Galen (Berryman, 2012; Tipton, 2014a, b). The latter work is credited with introducing the medical discussion of the so-called six non naturals (*res non naturales*); that is, things not innate: air, food and beverages, sleep and wakefulness, movement and rest, excretion, and mood (Jarcho, 1970). Avicenna’s *Poem on Medicine* enumerates recommendations corresponding to these six domains (Krueger, 1963). Similar considerations can be found in Maimonides’ treatise on hygiene called Regimen of Good Health (*Regimen sanitatis*). Similar views were echoed by German humanist philosopher Nicolaus Cusanus in the thirteenth century (Grmek, 1957, 64). These ideas formed a corpus of proto-hygienic norms of self-care ensuring longer and healthier lives, which have reappeared in various forms through modern times. The Italian humanistic tradition particularly contributed to the widespread propagation of such *rules of good health* (Gilleard, 2013). With its emphasis on virtuous conduct in both public and personal life, the humanistic tradition stimulated the publication of numerous treatises, or rather, practical manuals, on all aspects of life, such as running a family (see Leon Battista Alberti’s four books *Della Famiglia, 1443*), conducting oneself with measure in mundane circumstances (like *Il Libro del Cortigiano* by Baldassarre Castiglione, 1528), and physical exercise (as in *De Arte Gymnastica* written by Girolamo Mercuriale in 1569). Within this tradition, physician Gabriele Zerbi wrote his *Gerontocomia* (1489) on the care of the aged—a book that was recently rediscovered as a precursor of present-day literature on successful ageing (Gilleard, 2013; Katz & Calasanti, 2015). While Zerbi draws heavily on Galen’s non naturals, Marsilio Ficino, in his *De Triplici Vita* (The Book of Life), published in Florence the same year as Zerbi’s *Gerontocomia*, blends medicine, magic, and astrology to give advice on how to preserve health and prolong life. Of a less esoteric character, in the fifteen century Venetian nobleman Alvise Cornaro wrote his *Discourses on the Sober Life* (1558) in which, drawing on his own experience as a healthy elder, he criticized negative views of old age, and recommended the adoption of salutogenic habits, especially in terms of food consumption, in order to live longer and preserve good health over time (Cornaro, 1951; Howell, 1987). Both Ficino’s book and Cornaro’s *discourses* became extremely popular, as evidenced by their numerous translations and multiple editions (Gilleard, 2013; Tarabocchia Canavero, 1977).

Some 200 years later, in 1768, Linnaeus published under his name a dissertation by one of his students (Johannes Grysselius)—a common practice at that time—on how to preserve a sound constitution by intervening on the six non naturals, by means of healthy habits.

This ancient medical wisdom is not centred around any specific type of condition, but rather on the objective of ageing in good health. By way of illustration, chapter four of Maimonides’ Regimen consists of “advice that is beneficial in general and in particular for the healthy and for the sick” (Bar-Sela et al., 1964, 27). This approach stresses self-management and control over one’s health trajectory, without reference to a specific type of illness, with the aim of preserving well-being throughout all phases of life. This epistemological and practical stance echoes long-held views in scholastic medicine, from Galen to Avicenna, regarding health preservation as one of the two aims of medicine; the other being treating the sick. What I have called ground-state prevention reiterates the disease-agnostic character of these ancient medical ideas.

## Conclusion

Research into the biological and behavioral determinants of healthy longevity represents one of the most promising avenues of present-day biomedical research and public health. At the roots of the quest for improving the human health span lies an emerging explanatory framework about ageing itself. In this paper, I have provided some historico-philosophical clarifications on such biomedical understandings of ageing. I have illustrated an explanatory framework that, based on demographic, epidemiological, and molecular data, stresses the functional plasticity of the ageing process. Moreover, I have shown that a specific understanding of preventive medicine is associated with this explanatory framework. I have proposed to conceptualize it as ground-state prevention, and to view it in light of ancient medical ideas about the preservation of health throughout life.

Historical and philosophical clarification of various explanatory frameworks of ageing is important in its own right, and represents a necessary condition for exploring the normative implications intrinsic to present-day biomedical discourse about ageing. In particular, the critical appraisal of the different models of care, prevention, and health promotion cannot overlook their epistemological underpinnings. Failure to trace the conceptual lines linking models of ageing and models of care represents an impediment to the careful analysis of ethical, philosophical, and sociological issues around ageing.

Regarding ageing as a dynamic process, open to different functional and biological outcomes and amenable to preventive intervention, has numerous normative implications. In particular, the plasticity of ageing lends support to the idea that people should strive for a longer and healthier life—a debated issue in moral philosophy since ancient times (Seneca, 2004; Wareham, 2021). Moreover, seeing ageing in terms of modifiable functional trajectories that depend, at least in part, on individual lifestyle, seems to incorporate tacit ethical assumptions regarding personal responsibility for the way individuals age. These considerations impinge on ultimately political discussions regarding the role and possibly the limits of healthcare. Of equal importance, they have implications for the cultural attributes of ageing. In this respect there is a risk that those who fail to comply with health promotion recommendations regarding the management of age-related health risks will be stigmatized or discriminated against.

Increasing the proportion of elders that reach old age in good shape is a legitimate and laudable public health goal. However, attention must be paid to the distribution of opportunities for healthy ageing within a population, as well as globally. Given the combined effects of socio-economic inequity, systemic racism, and persistent disparities linked to the historical legacy of colonialism, the prospects for healthy ageing are very different for people living in different countries and belonging to different social and ethnic groups.

Finally, any behavioral or pharmacological intervention aimed at improving the functional trajectory of ageing implies reflection on the value of typically young forms of functioning (Jecker, 2021).

Our biomedical understanding of ageing can be attributed to scientific insights into the biology of ageing, as well as epidemiological and experimental evidence about its plasticity. But as we have seen, value-laden assumptions can build within this explanatory framework, determining how it will ultimately affect people’s lives in tangible ways. Research is needed to further understand those assumptions and assess them in light of robust ethical principles. Furthermore, specific policy efforts should be directed towards ensuring that opportunities for healthy ageing are fairly distributed and that ground-state prevention strategies are equitably offered to all.

## Footnotes

1 This essay has been partially reprinted in (M. Grmek 2018).

2 Throughout the paper I use the term ‘disability’ mostly to indicate the age-dependent decline or loss of functional abilities. In geriatrics, this meaning of the term is captured by the notion of functional status (and decline thereof), indicating the measurable effect of multimorbidity, polypharmacy and physical warn-out on an individual’s on a number of functional domains, such as: gait, balance and risk of falls, cognition, nutrition, bladder control, affective status and social function (Bernabei et al., 2000).

3 Reviewed in (Braeckman and Vanfleteren 2007).
